# Supplementary material for: Asymmetric Synthesis of Quaternary Hydantoins via a Palladium-Catalyzed Aza-Heck Cyclization
Source: J Am Chem Soc. 2025 Nov 14;147(49):44692–8. doi: 10.1021/jacs.5c16022 (PMC12703750; doi:10.1021/jacs.5c16022)
Supplement: Supplementary file 2 [file ja5c16022_si_002.zip › All NMR FID Files/13/13_CarbonNMR/pdata/1/email_TDI01-198 xterize again 3_10_1.pdf]

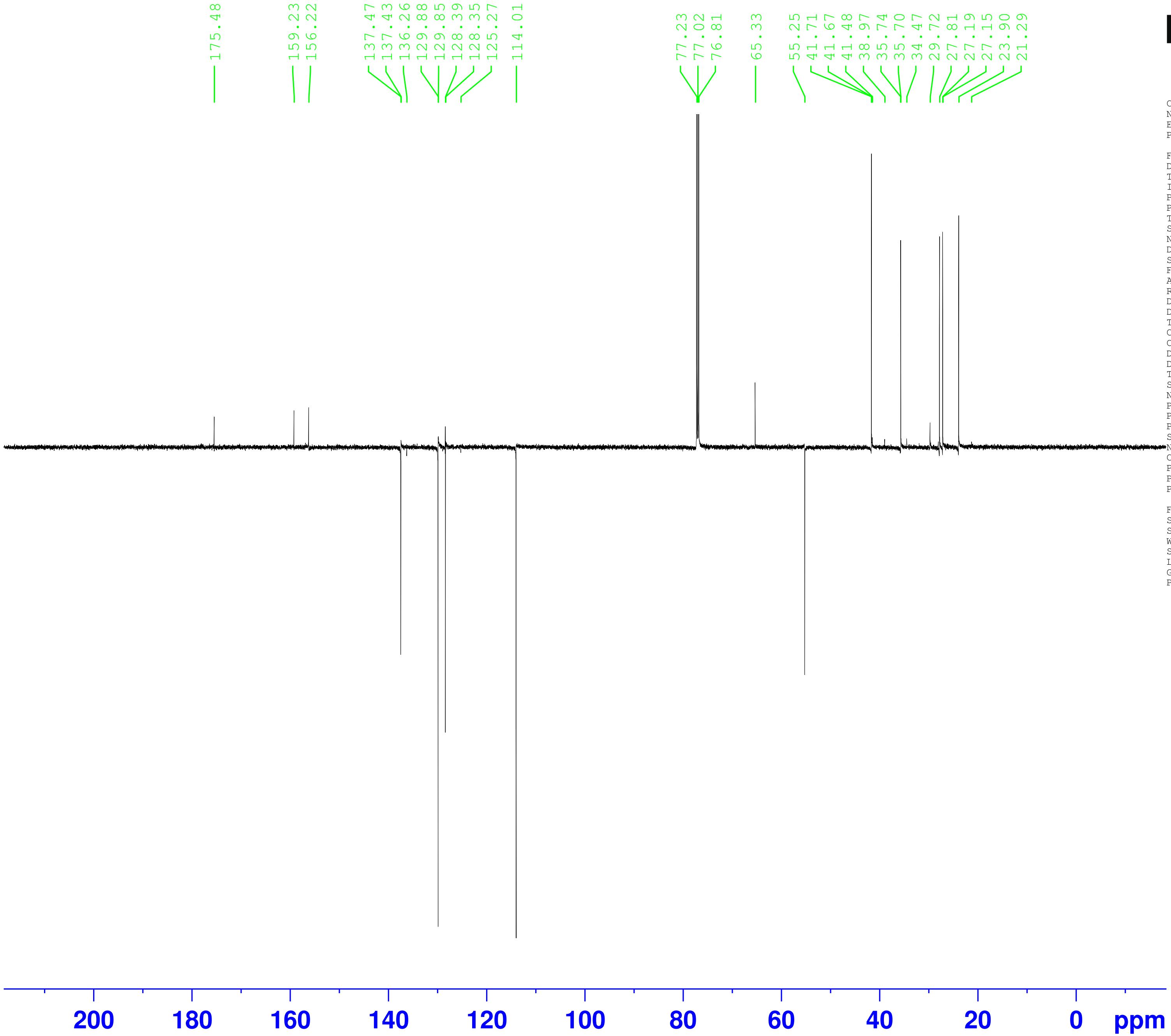

Current Data Parameters

NAME	TDI01-198 xterize again 3	
EXPNO	10	
PROCNO	1	

F2 - Acquisition Parameters

Date_	20250220	
Time	1.06	h
INSTRUM	Avance Neo 600	at University of Delaware
PROBHD	Z185170_0001 (	
PULPROG	jmod	
TD	65536	
SOLVENT	CDCl3	
NS	1024	
DS	4	
SWH	35714.286	Hz
FIDRES	1.089913	Hz
AQ	0.9175040	sec
RG	101	
DW	14.000	usec
DE	19.25	usec
TE	298.0	K
CNST2	145.0000000	
CNST11	1.0000000	
D1	2.00000000	sec
D20	0.00689655	sec
TD0	1	
SFO1	150.9178988	MHz
NUC1	13C	
P1	11.98	usec
P2	23.96	usec
PLW1	106.00000000	W
SFO2	600.1324005	MHz
NUC2	1H	
CPDPRG[2	waltz65	
PCPD2	70.00	usec
PLW2	6.90000010	W
PLW12	0.09170600	W

F2 - Processing parameters

SI	32768	
SF	150.9028085	MHz
WDW	EM	
SSB	0	
LB	1.00	Hz
GB	0	
PC	1.40	
